# *De Novo* Genome Assembly of a *Plasmodium falciparum* NF54 Clone Using Single-Molecule Real-Time Sequencing

**DOI:** 10.1128/genomeA.01479-17

**Published:** 2018-02-01

**Authors:** Jessica M. Bryant, Sebastian Baumgarten, Audrey Lorthiois, Christine Scheidig-Benatar, Aurélie Claës, Artur Scherf

**Affiliations:** a Unité de Biologie des Interactions Hôte-Parasite, Institut Pasteur, Paris, France; b INSERM U1201, Paris, France; c CNRS ERL9195, Paris, France; d Center for Production and Infection of Anopheles (CEPIA), Institut Pasteur, Paris, France

## Abstract

*Plasmodium falciparum* is the species of human malaria parasite that causes the most severe form of the disease. Here, we used single-molecule real-time (SMRT) sequencing technology from Pacific Biosciences (PacBio) to sequence, assemble *de novo*, and annotate the genome of a *P. falciparum* NF54 clone.

## GENOME ANNOUNCEMENT

*Plasmodium falciparum* is a protozoan parasite with a complex life cycle in human and mosquito hosts (1). Its 23-Mb haploid genome is extremely AT rich (~80%) and contains stretches of highly repetitive sequences, especially in telomeric and subtelomeric regions. As genome editing techniques advance, the need has arisen for a complete and accurate genome sequence of laboratory strains used to investigate pathogenesis. The genome of the *P. falciparum* 3D7 strain, which is widely used to study the intraerythrocytic life cycle *in vitro*, has been sequenced and annotated (2). 3D7 is a clone of the NF54 isolate, which is believed to have originated in Africa (3, 4). However, 3D7 produces fewer gametocytes under *in vitro* culture conditions than the original NF54 strain (5). Here, we present the nuclear genome sequence and annotation of a new NF54 clone. We have shown that this clone can be easily grown in culture and transmitted to the mosquito, producing an amount of salivary gland sporozoites similar to that of its parent NF54 strain. This strain and its genome sequence will be useful for performing functional studies of gametocytogenesis, mosquito transmission, and mosquito stage development.

Late-stage parasites were grown in 6 ml of concentrated blood at 3.5% parasitemia and enriched by Plasmagel flotation. DNA was extracted with the Genomic-tip 500/G kit (catalog number 10262; Qiagen) and further purified with phenol-chloroform. Library preparation and sequencing were performed by GATC Biotech (Constance, Germany) on a PacBio RS II sequencer, with an average read length of 16,291 bp and genome coverage of 100× postfiltering. The same genomic DNA was sonicated, and libraries were prepared with the NEBNext Ultra II DNA library preparation kit (catalog number E7645S; New England Biolabs) and sequenced with a NextSeq 500 platform (Illumina) with an average genome coverage of 600×.

The genome was assembled using the PacBio RS_Assembly_HGAP.3 protocol (included in SMRT Portal version 2.3.0), with default settings and a target genome size of 23 Mb (6). Scaffolding of the initial assembly was performed using pyScaf (https://github.com/lpryszcz/pyScaf) and the 3D7 genome as a reference. The resulting gaps were closed with GapFiller (7) using the Illumina short reads. The assembly was further polished with the PacBio reads using BLASR (8) and Quiver (6). Illumina short reads were subsequently mapped to the assembly using Bowtie2 (9), and read alignments were used to fix remaining single-nucleotide polymorphisms (SNPs), short indels, and breaks using Pilon (10).

The final assembly is composed of 19 contigs, with a total size of 23,435,585 bases and an average GC content of 19.34%. All 14 chromosomes found in the 3D7 strain are present in our NF54 assembly, with 9 contigs representing putatively full-length chromosomes. The remaining 5 chromosomes are assembled in no more than two contigs per chromosome.

A LiftOver annotation was performed using FLO (https://github.com/wurmlab/flo) with the curated 3D7 annotation provided at PlasmoDB version 32 (http://plasmodb.org) (11). In total, 5,587 loci were identified, of which 5,015 loci are protein coding, 399 loci are putative pseudogenes, and 173 loci are non-protein-coding RNA. All multigene families associated with antigenic variation were annotated, including *var* (59 genes), *rifin* (158 genes), and *stevor* (31 genes).

### Accession number(s).

This whole-genome shotgun project has been deposited at DDBJ/ENA/GenBank under the accession number NYMT00000000. The version described in this paper is version NYMT01000000.
